# Supplementary material for: Hot spot prediction in protein-protein interactions by an ensemble system
Source: BMC Syst Biol. 2018 Dec 31;12(Suppl 9):132. doi: 10.1186/s12918-018-0665-8 (PMC6311905; doi:10.1186/s12918-018-0665-8)
Supplement: Supplementary file 2 — All relevant MATLAB code. A simple Matlab implement of our predictor. (ZIP 19,742 kb) [file 12918_2018_665_MOESM2_ESM.zip › Additional file 2/Read ASA/ASA website/dbMPIKT/4JEUB.html]

 NetSurfP - Protein Surface Accessibility and Secondary Structure Predictions

  

|  |  |  |
| --- | --- | --- |
|  |  | NetSurfP - Protein Surface Accessibility and Secondary Structure Predictions  Technical University of Denmark |

  


---

```
# For publication of results, please cite:
# A generic method for assignment of reliability scores applied to solvent accessibility predictions.
# Bent Petersen, Thomas Nordahl Petersen, Pernille Andersen, Morten Nielsen and Claus Lundegaard
# BMC Structural Biology 2009, 9:51 doi:10.1186/1472-6807-9-51
#
# Column 1: Class assignment - B for buried or E for Exposed - Threshold: 25% exposure, but not based on RSA
# Column 2: Amino acid
# Column 3: Sequence name
# Column 4: Amino acid number
# Column 5: Relative Surface Accessibility - RSA
# Column 6: Absolute Surface Accessibility
# Column 7: Z-fit score for RSA prediction
# Column 8: Probability for Alpha-Helix
# Column 9: Probability for Beta-strand
# Column 10: Probability for Coil
E K  4JEUB                  1    0.709 145.738   0.996   0.003   0.003   0.994
E D  4JEUB                  2    0.487  70.220  -1.116   0.181   0.016   0.803
B R  4JEUB                  3    0.289  66.089   0.621   0.717   0.014   0.269
B T  4JEUB                  4    0.245  33.940   0.011   0.858   0.002   0.139
E Q  4JEUB                  5    0.518  92.568   0.733   0.923   0.002   0.076
E E  4JEUB                  6    0.322  56.271   0.424   0.923   0.002   0.076
B L  4JEUB                  7    0.121  22.192   0.231   0.879   0.010   0.111
E R  4JEUB                  8    0.422  96.638   0.685   0.802   0.014   0.185
E T  4JEUB                  9    0.489  67.838  -0.405   0.600   0.003   0.397
E A  4JEUB                 10    0.409  45.116  -0.879   0.406   0.004   0.590
E K  4JEUB                 11    0.412  84.687  -0.856   0.257   0.016   0.727
E D  4JEUB                 12    0.582  83.881  -0.987   0.181   0.016   0.803
E S  4JEUB                 13    0.468  54.861  -0.983   0.115   0.016   0.868
E D  4JEUB                 14    0.634  91.374  -0.648   0.058   0.017   0.925
E D  4JEUB                 15    0.605  87.209  -0.801   0.115   0.016   0.868
E D  4JEUB                 16    0.491  70.782  -1.673   0.181   0.016   0.803
E D  4JEUB                 17    0.585  84.255  -0.912   0.184   0.043   0.773
E D  4JEUB                 18    0.577  83.117  -0.484   0.191   0.086   0.723
E V  4JEUB                 19    0.390  59.882  -1.230   0.191   0.086   0.723
E T  4JEUB                 20    0.492  68.268  -1.261   0.113   0.087   0.800
E V  4JEUB                 21    0.318  48.923  -1.723   0.118   0.150   0.732
E T  4JEUB                 22    0.314  43.538  -1.642   0.056   0.142   0.802
E V  4JEUB                 23    0.363  55.778  -1.757   0.052   0.084   0.864
E D  4JEUB                 24    0.491  70.796  -1.480   0.018   0.047   0.935
E R  4JEUB                 25    0.451 103.256  -1.341   0.018   0.019   0.964
E D  4JEUB                 26    0.566  81.618  -1.549   0.058   0.017   0.925
E R  4JEUB                 27    0.636 145.575  -1.175   0.053   0.005   0.942
E F  4JEUB                 28    0.324  65.027  -0.687   0.053   0.005   0.942
B M  4JEUB                 29    0.131  26.153   0.505   0.694   0.003   0.303
E D  4JEUB                 30    0.651  93.751   0.932   0.923   0.002   0.076
E E  4JEUB                 31    0.516  90.110   1.680   0.970   0.001   0.030
B F  4JEUB                 32    0.039   7.867  -0.086   0.970   0.001   0.030
B F  4JEUB                 33    0.147  29.523   0.632   0.970   0.001   0.030
E E  4JEUB                 34    0.638 111.406   1.608   0.970   0.001   0.030
E Q  4JEUB                 35    0.272  48.633   1.672   0.970   0.001   0.030
B V  4JEUB                 36    0.023   3.581   0.594   0.970   0.001   0.030
E E  4JEUB                 37    0.503  87.804   1.870   0.970   0.001   0.030
E E  4JEUB                 38    0.512  89.394   2.168   0.970   0.001   0.030
B I  4JEUB                 39    0.018   3.237   1.427   0.970   0.001   0.030
E R  4JEUB                 40    0.268  61.258   1.171   0.970   0.001   0.030
E G  4JEUB                 41    0.554  43.576   1.662   0.970   0.001   0.030
B F  4JEUB                 42    0.211  42.368   1.418   0.988   0.000   0.012
B I  4JEUB                 43    0.027   4.940   0.598   0.988   0.000   0.012
E D  4JEUB                 44    0.469  67.540   1.835   0.988   0.000   0.012
E K  4JEUB                 45    0.456  93.840   2.461   0.988   0.000   0.012
B I  4JEUB                 46    0.028   5.161   0.668   0.970   0.001   0.030
E A  4JEUB                 47    0.325  35.760   1.106   0.970   0.001   0.030
E E  4JEUB                 48    0.567  99.090   1.644   0.970   0.001   0.030
B N  4JEUB                 49    0.207  30.232   1.203   0.970   0.001   0.030
B V  4JEUB                 50    0.049   7.516   0.539   0.970   0.001   0.030
E E  4JEUB                 51    0.531  92.783   1.660   0.970   0.001   0.030
E E  4JEUB                 52    0.435  75.995   2.346   0.970   0.001   0.030
B V  4JEUB                 53    0.026   4.012   0.741   0.970   0.001   0.030
E K  4JEUB                 54    0.356  73.126   1.515   0.988   0.000   0.012
E R  4JEUB                 55    0.589 134.881   1.826   0.988   0.000   0.012
B K  4JEUB                 56    0.231  47.414   1.179   0.970   0.001   0.030
B H  4JEUB                 57    0.148  26.867   0.374   0.970   0.001   0.030
E S  4JEUB                 58    0.535  62.690   0.802   0.923   0.002   0.076
E A  4JEUB                 59    0.520  57.337   0.850   0.858   0.002   0.139
E I  4JEUB                 60    0.305  56.443  -0.185   0.782   0.003   0.216
E L  4JEUB                 61    0.302  55.351  -0.777   0.406   0.004   0.590
E A  4JEUB                 62    0.483  53.194  -1.043   0.109   0.005   0.886
E S  4JEUB                 63    0.403  47.278  -0.857   0.053   0.005   0.942
E P  4JEUB                 64    0.384  54.546  -1.705   0.016   0.005   0.979
E N  4JEUB                 65    0.602  88.191  -1.000   0.016   0.005   0.979
E P  4JEUB                 66    0.511  72.511  -1.025   0.053   0.005   0.942
E D  4JEUB                 67    0.487  70.191  -0.770   0.321   0.003   0.675
E E  4JEUB                 68    0.659 115.075   0.617   0.858   0.002   0.139
E K  4JEUB                 69    0.608 125.086   1.037   0.923   0.002   0.076
B T  4JEUB                 70    0.096  13.301   0.158   0.923   0.002   0.076
E K  4JEUB                 71    0.357  73.373   1.041   0.970   0.001   0.030
E E  4JEUB                 72    0.653 114.009   1.210   0.970   0.001   0.030
E E  4JEUB                 73    0.392  68.413   1.598   0.988   0.000   0.012
B L  4JEUB                 74    0.040   7.287   0.278   0.988   0.000   0.012
E E  4JEUB                 75    0.505  88.276   1.137   0.988   0.000   0.012
E E  4JEUB                 76    0.538  93.954   1.677   0.970   0.001   0.030
B L  4JEUB                 77    0.099  18.164   0.906   0.970   0.001   0.030
B M  4JEUB                 78    0.141  28.294   0.811   0.988   0.000   0.012
E S  4JEUB                 79    0.553  64.847   1.317   0.988   0.000   0.012
E D  4JEUB                 80    0.394  56.747   2.004   0.988   0.000   0.012
B I  4JEUB                 81    0.017   3.108   1.127   0.988   0.000   0.012
E K  4JEUB                 82    0.321  65.947   1.409   0.988   0.000   0.012
E K  4JEUB                 83    0.565 116.179   1.514   0.970   0.001   0.030
B T  4JEUB                 84    0.157  21.748   0.741   0.988   0.000   0.012
B A  4JEUB                 85    0.029   3.240  -0.378   0.988   0.000   0.012
E N  4JEUB                 86    0.509  74.591   1.838   0.988   0.000   0.012
E K  4JEUB                 87    0.451  92.853   1.926   0.988   0.000   0.012
B V  4JEUB                 88    0.021   3.166   0.873   0.988   0.000   0.012
E R  4JEUB                 89    0.330  75.639   1.306   0.988   0.000   0.012
E S  4JEUB                 90    0.549  64.390   1.498   0.988   0.000   0.012
B K  4JEUB                 91    0.205  42.107   1.425   0.988   0.000   0.012
B L  4JEUB                 92    0.036   6.555   0.093   0.970   0.001   0.030
E K  4JEUB                 93    0.517 106.450   1.915   0.970   0.001   0.030
E S  4JEUB                 94    0.463  54.299   2.172   0.970   0.001   0.030
B I  4JEUB                 95    0.023   4.163   1.148   0.970   0.001   0.030
E E  4JEUB                 96    0.373  65.216   1.552   0.970   0.001   0.030
E Q  4JEUB                 97    0.579 103.481   1.557   0.970   0.001   0.030
B S  4JEUB                 98    0.229  26.897   0.356   0.970   0.001   0.030
B I  4JEUB                 99    0.101  18.630  -0.741   0.923   0.002   0.076
E E  4JEUB                100    0.646 112.839   0.672   0.923   0.002   0.076
E Q  4JEUB                101    0.611 109.125   0.307   0.782   0.003   0.216
E E  4JEUB                102    0.301  52.602  -0.947   0.600   0.003   0.397
E E  4JEUB                103    0.631 110.201  -0.661   0.406   0.004   0.590
E G  4JEUB                104    0.715  56.310  -0.486   0.246   0.004   0.750
E L  4JEUB                105    0.471  86.222  -0.798   0.115   0.016   0.868
E N  4JEUB                106    0.652  95.409  -1.263   0.058   0.017   0.925
E R  4JEUB                107    0.566 129.683  -1.332   0.058   0.017   0.925
E S  4JEUB                108    0.479  56.115  -1.979   0.053   0.005   0.942
E S  4JEUB                109    0.547  64.073  -0.634   0.406   0.004   0.590
E A  4JEUB                110    0.501  55.265  -1.045   0.923   0.002   0.076
E D  4JEUB                111    0.361  52.063  -0.984   0.923   0.002   0.076
E L  4JEUB                112    0.358  65.568  -0.516   0.970   0.001   0.030
E R  4JEUB                113    0.415  95.081   0.546   0.970   0.001   0.030
B I  4JEUB                114    0.243  45.029   0.269   0.970   0.001   0.030
B R  4JEUB                115    0.258  58.990   0.686   0.988   0.000   0.012
E K  4JEUB                116    0.432  88.945   1.063   0.988   0.000   0.012
B T  4JEUB                117    0.249  34.481   0.951   0.988   0.000   0.012
B Q  4JEUB                118    0.091  16.199   0.755   0.988   0.000   0.012
B H  4JEUB                119    0.205  37.217   0.725   0.988   0.000   0.012
E S  4JEUB                120    0.483  56.643   1.019   0.988   0.000   0.012
E T  4JEUB                121    0.343  47.616   1.534   0.988   0.000   0.012
B L  4JEUB                122    0.037   6.775  -0.070   0.988   0.000   0.012
E S  4JEUB                123    0.252  29.593   1.419   0.988   0.000   0.012
E R  4JEUB                124    0.565 129.316   1.335   0.988   0.000   0.012
E K  4JEUB                125    0.339  69.773   1.978   0.988   0.000   0.012
B F  4JEUB                126    0.024   4.877   0.833   0.988   0.000   0.012
E V  4JEUB                127    0.352  54.179   2.004   0.988   0.000   0.012
E E  4JEUB                128    0.548  95.683   1.649   0.988   0.000   0.012
B V  4JEUB                129    0.051   7.854   0.666   0.988   0.000   0.012
B M  4JEUB                130    0.075  14.927  -0.353   0.970   0.001   0.030
E S  4JEUB                131    0.525  61.542   1.289   0.988   0.000   0.012
E E  4JEUB                132    0.429  74.964   1.553   0.988   0.000   0.012
B Y  4JEUB                133    0.053  11.347   0.318   0.970   0.001   0.030
E N  4JEUB                134    0.348  51.020   1.356   0.988   0.000   0.012
E A  4JEUB                135    0.559  61.591   1.727   0.988   0.000   0.012
B T  4JEUB                136    0.133  18.489   1.087   0.988   0.000   0.012
B Q  4JEUB                137    0.102  18.199   0.596   0.988   0.000   0.012
E S  4JEUB                138    0.546  63.944   1.566   0.970   0.001   0.030
E D  4JEUB                139    0.467  67.251   1.874   0.988   0.000   0.012
B Y  4JEUB                140    0.089  18.955   0.724   0.988   0.000   0.012
E R  4JEUB                141    0.338  77.379   1.226   0.988   0.000   0.012
E E  4JEUB                142    0.634 110.742   1.330   0.988   0.000   0.012
E R  4JEUB                143    0.464 106.187   1.635   0.988   0.000   0.012
B C  4JEUB                144    0.116  16.343   0.710   0.970   0.001   0.030
E K  4JEUB                145    0.451  92.853   0.957   0.970   0.001   0.030
E G  4JEUB                146    0.615  48.408   0.953   0.970   0.001   0.030
E R  4JEUB                147    0.354  80.952   1.123   0.970   0.001   0.030
B I  4JEUB                148    0.157  29.045  -0.191   0.970   0.001   0.030
E Q  4JEUB                149    0.511  91.265   0.533   0.970   0.001   0.030
E R  4JEUB                150    0.415  94.920   0.351   0.938   0.007   0.055
E Q  4JEUB                151    0.295  52.758  -0.238   0.879   0.010   0.111
E L  4JEUB                152    0.318  58.226  -0.404   0.802   0.014   0.185
E E  4JEUB                153    0.580 101.413   0.098   0.717   0.014   0.269
E I  4JEUB                154    0.301  55.648  -1.172   0.430   0.016   0.555
E T  4JEUB                155    0.478  66.243  -1.455   0.257   0.016   0.727
E G  4JEUB                156    0.548  43.104  -1.482   0.115   0.016   0.868
E R  4JEUB                157    0.533 121.943  -1.431   0.058   0.017   0.925
E T  4JEUB                158    0.608  84.343  -2.079   0.058   0.017   0.925
B T  4JEUB                159    0.265  36.714  -1.699   0.058   0.017   0.925
E T  4JEUB                160    0.488  67.644  -0.777   0.115   0.016   0.868
E S  4JEUB                161    0.590  69.195  -1.413   0.694   0.003   0.303
E E  4JEUB                162    0.625 109.153  -0.859   0.782   0.003   0.216
E E  4JEUB                163    0.399  69.740  -1.036   0.782   0.003   0.216
B L  4JEUB                164    0.228  41.710  -0.731   0.879   0.010   0.111
E E  4JEUB                165    0.575 100.470  -0.297   0.802   0.014   0.185
E D  4JEUB                166    0.556  80.163  -0.084   0.802   0.014   0.185
B M  4JEUB                167    0.275  54.947  -1.250   0.717   0.014   0.269
E L  4JEUB                168    0.325  59.507  -1.425   0.522   0.016   0.462
E E  4JEUB                169    0.546  95.474  -1.332   0.339   0.016   0.645
E S  4JEUB                170    0.496  58.178  -1.373   0.257   0.016   0.727
E G  4JEUB                171    0.509  40.098  -1.979   0.257   0.016   0.727
E N  4JEUB                172    0.513  75.089  -1.670   0.339   0.016   0.645
E P  4JEUB                173    0.445  63.075  -1.576   0.782   0.003   0.216
E A  4JEUB                174    0.502  55.320  -1.272   0.923   0.002   0.076
E I  4JEUB                175    0.392  72.502  -0.871   0.938   0.007   0.055
B F  4JEUB                176    0.240  48.108  -0.507   0.938   0.007   0.055
E A  4JEUB                177    0.345  37.986  -0.743   0.938   0.007   0.055
E S  4JEUB                178    0.412  48.251  -1.109   0.879   0.010   0.111
E G  4JEUB                179    0.421  33.141  -0.793   0.782   0.003   0.216
E I  4JEUB                180    0.335  61.994  -0.577   0.782   0.003   0.216
E I  4JEUB                181    0.441  81.511  -0.732   0.694   0.003   0.303
E M  4JEUB                182    0.456  91.226  -0.620   0.694   0.003   0.303
E D  4JEUB                183    0.551  79.414  -1.035   0.600   0.003   0.397
E S  4JEUB                184    0.428  50.103  -0.616   0.782   0.003   0.216
E S  4JEUB                185    0.616  72.172  -0.309   0.858   0.002   0.139
E I  4JEUB                186    0.422  78.014   0.243   0.923   0.002   0.076
B S  4JEUB                187    0.259  30.402  -0.100   0.923   0.002   0.076
E K  4JEUB                188    0.522 107.375   1.108   0.970   0.001   0.030
E Q  4JEUB                189    0.504  90.032   0.390   0.970   0.001   0.030
B A  4JEUB                190    0.183  20.134   0.292   0.970   0.001   0.030
B L  4JEUB                191    0.232  42.498   0.161   0.970   0.001   0.030
E S  4JEUB                192    0.545  63.874   1.046   0.970   0.001   0.030
E E  4JEUB                193    0.367  64.045   1.649   0.970   0.001   0.030
B I  4JEUB                194    0.085  15.651   1.049   0.970   0.001   0.030
E E  4JEUB                195    0.448  78.248   1.580   0.970   0.001   0.030
E T  4JEUB                196    0.560  77.686   1.263   0.970   0.001   0.030
B R  4JEUB                197    0.200  45.869   0.689   0.970   0.001   0.030
B H  4JEUB                198    0.280  50.877   0.249   0.970   0.001   0.030
E S  4JEUB                199    0.598  70.027   1.465   0.970   0.001   0.030
B E  4JEUB                200    0.259  45.282   0.820   0.970   0.001   0.030
B I  4JEUB                201    0.058  10.637   0.346   0.970   0.001   0.030
E I  4JEUB                202    0.437  80.919   1.557   0.970   0.001   0.030
E K  4JEUB                203    0.506 104.125   1.957   0.970   0.001   0.030
B L  4JEUB                204    0.053   9.723   0.789   0.970   0.001   0.030
B E  4JEUB                205    0.207  36.110   0.060   0.970   0.001   0.030
E N  4JEUB                206    0.507  74.152   1.154   0.970   0.001   0.030
B S  4JEUB                207    0.134  15.646   0.533   0.923   0.002   0.076
B I  4JEUB                208    0.030   5.513   0.374   0.970   0.001   0.030
E R  4JEUB                209    0.366  83.814   1.389   0.970   0.001   0.030
E E  4JEUB                210    0.326  57.022   1.159   0.970   0.001   0.030
B L  4JEUB                211    0.026   4.761   0.642   0.988   0.000   0.012
B H  4JEUB                212    0.222  40.400   0.606   0.988   0.000   0.012
E D  4JEUB                213    0.477  68.750   1.520   0.988   0.000   0.012
B M  4JEUB                214    0.046   9.225   0.925   0.988   0.000   0.012
B F  4JEUB                215    0.064  12.825   0.769   0.988   0.000   0.012
E M  4JEUB                216    0.373  74.677   1.373   0.988   0.000   0.012
B D  4JEUB                217    0.285  41.040   0.429   0.988   0.000   0.012
B M  4JEUB                218    0.038   7.504   0.305   0.988   0.000   0.012
B A  4JEUB                219    0.149  16.365  -0.294   0.988   0.000   0.012
E M  4JEUB                220    0.379  75.778   0.971   0.988   0.000   0.012
B L  4JEUB                221    0.068  12.378   1.306   0.970   0.001   0.030
B V  4JEUB                222    0.048   7.408   0.571   0.975   0.003   0.022
E E  4JEUB                223    0.424  74.038   1.252   0.938   0.007   0.055
E S  4JEUB                224    0.462  54.088   0.691   0.802   0.014   0.185
B Q  4JEUB                225    0.166  29.737   0.069   0.694   0.003   0.303
E G  4JEUB                226    0.347  27.301  -0.140   0.600   0.003   0.397
E E  4JEUB                227    0.559  97.640   0.692   0.782   0.003   0.216
B M  4JEUB                228    0.166  33.137   0.853   0.879   0.010   0.111
B I  4JEUB                229    0.041   7.548   0.378   0.938   0.007   0.055
E D  4JEUB                230    0.448  64.528   1.139   0.938   0.007   0.055
E R  4JEUB                231    0.342  78.226   1.873   0.975   0.003   0.022
B I  4JEUB                232    0.023   4.310   1.165   0.970   0.001   0.030
B E  4JEUB                233    0.239  41.736   0.532   0.970   0.001   0.030
E Y  4JEUB                234    0.532 113.710   1.348   0.970   0.001   0.030
B N  4JEUB                235    0.158  23.102   0.714   0.970   0.001   0.030
B V  4JEUB                236    0.039   6.010   0.001   0.970   0.001   0.030
E E  4JEUB                237    0.501  87.507   1.363   0.923   0.002   0.076
E H  4JEUB                238    0.511  92.878   1.788   0.858   0.002   0.139
B A  4JEUB                239    0.067   7.339  -1.441   0.782   0.003   0.216
E V  4JEUB                240    0.520  79.985   0.366   0.622   0.015   0.363
E D  4JEUB                241    0.717 103.276   0.938   0.339   0.016   0.645
E Y  4JEUB                242    0.532 113.624  -0.554   0.016   0.005   0.979

Explain the output.  Go back.
```

---
